# Researching what matters to improve chronic pain care in Canada: A priority-setting partnership process to support patient-oriented research

**DOI:** 10.1080/24740527.2018.1433959

**Published:** 2018-07-19

**Authors:** Patricia Poulin, Yaadwinder Shergill, Heather Romanow, Jason W. Busse, Christine T. Chambers, Lynn Cooper, Paula A. Forgeron, Anita Olsen Harper, Maria Hudspith, Alfonso Iorio, Chitra Lalloo, Carley Ouellette, Rosalind Robertson, Sandy Smeenk, Bonnie Stevens, Jennifer Stinson

**Affiliations:** a The Ottawa Hospital Research Institute, The Ottawa Hospital Pain Clinic, Ottawa, ON, Canada; b School of Psychology, Faculty of Social Sciences, Department of Anesthesiology and Pain Medicine, Faculty of Medicine, University of Ottawa, Ottawa, ON, Canada; c Centre for Collaborative Health, Oakville, ON, Canada; d The Ottawa Hospital Research Institute, Ottawa, ON, Canada; e Department of Anesthesia, McMaster University, Hamilton, ON, Canada; f Department of Health Research Methods, Evidence and Impact, The Michael G. DeGroote Institute for Pain Research and Care, McMaster University Hamilton Health Sciences Centre, Hamilton, ON, Canada; g Pediatrics and Psychology & Neuroscience, Neuroscience, Dalhousie University and IWK Health Centre, Halifax, NS, Canada; h Canadian Pain Coalition, Oshawa, ON, Canada; i School of Nursing, University of Ottawa, Ottawa, ON, Canada; j Faculty of Medicine, Dalhousie University; k Children’s Hospital of Easter Ontario Research Institute, Ottawa, ON, Canada; l Independent Researcher, Ottawa, ON, Canada; m Pain BC, Vancouver, BC, Canada; n Department of Medicine; o Health Information Research Unit; p Hemophilia Clinic, McMaster University, Hamilton, ON, Canada; q Improving Outcomes in Child Health Through Technology (iOUCH) Lab; r Child Health Evaluative Sciences, The Hospital for Sick Children, Institute of Health Policy, Management and EvaluationToronto, ON, Canada; s Child Health Evaluative Sciences, Toronto, ON, Canada; t School of Nursing, The Peter Gilgan Centre for Research and Learning, Toronto, ON, Canada; u School of Nursing, McMaster University, Hamilton, ON, Canada; v Toronto, ON, Canada; w The ILC Chronic Pain and Ehlers Danlos Charitable Foundation, Oakville, ON, Canada; x University of Toronto Centre for the Study of Pain; y Research Institute, The Hospital for Sick Children, Toronto, ON, Canada; z Chronic Pain Program, The Hospital for Sick Children

**Keywords:** chronic pain, Delphi, patient-oriented research, priority-setting partnership

## Abstract

**Background:**

Chronic pain affects more than 6 million Canadians. Patients need to be involved in setting research priorities to ensure a focus on areas important to those who will be most impacted by the results.

**Aims:**

The aim of this study was to leverage patient experiences to identify chronic pain research priorities in Canada.

**Method:**

The process was informed by the James Lind Alliance. After gathering an exhaustive list of questions using surveys, town hall meetings, interviews, and social media consultations, we used a computerized Delphi with four successive iterations to select the final list of research priorities. The final Delphi round was conducted by a panel of ten patients living with chronic pain and ten clinicians from different disciplines.

**Results:**

We received more than 5000 suggestions from 1500 people. The Delphi process led to the identification of 14 questions fitting under the following 4 themes: (1) improving knowledge and competencies in chronic pain; (2) improving patient-centered chronic pain care; (3) preventing chronic pain and reducing associated symptoms; and (4) improving access to and coordination of patient-centered chronic pain care. Challenges included the issue of chronic pain being ubiquitous to many diseases, leading to many initial suggestions focusing on these diseases. We also identified the need for further engagement efforts with marginalized groups in order to validate the priorities identified or identify different sets of priorities specific to these groups.

**Conclusion:**

The priorities identified can guide patient-oriented chronic pain research to ultimately improve the care offered to people living with chronic pain.

## RÉSUMÉ

**Contexte**: La douleur chronique affecte plus de six millions de Canadiens. Les patients doivent participer à l’établissement des priorités de recherche afin de s’assurer que l’accent soit mis sur les aspects les plus importants aux yeux de ceux qui seront les plus touchés par les résultats.

**Buts**: Tirer parti de l’expérience des patients afin d’établir les priorités de la recherche en matière de douleur chronique au Canada.

**Méthode**: Le processus s’est impiré de la James Lind Alliance. Après avoir dressé une liste exhaustive de questions à l’aide d’enquêtes, d’assemblées publiques, d’entrevues et de consultations via les médias sociaux, nous avons utilisé un processus Delphi informatisé comprenant quatre itérations successives afin de sélectionner la liste finale de priorités de recherche. Le dernier tour du processus Delphi a été mené par un panel composé de 10 patients souffrant de douleur chronique et de 10 cliniciens issus de différentes disciplines.

**Résultats**: Nous avons reçu plus de 5 000 suggestions provenant de 1 500 personnes. Le processus Delphi a permis de déterminer 14 questions qui relèvent des quatre thèmes suivants : (1) améliorer les connaissances et les compétences en matière de douleur chronique ; (2) améliorer les soins axés sur le patient pour contrer la douleur chronique; (3) prévenir la douleur chronique et réduire les symptômes qui y sont associés ; et (4) améliorer l’accès aux soins axés sur le patient pour contrer la douleur chronique et la coordination entre ces soins. Parmi les défis qui se sont présentés, on compte le fait que la douleur chronique soit omniprésente dans de nombreuses maladies, de sorte qu’un grand nombre des suggestions initialement formulées portaient sur ces maladies. Nous avons également déterminé que davantage d’efforts devaient être déployés pour assurer la participation des groupes marginalisés dans le but de valider les priorités déterminées ou déterminer d’autres priorités spécifiques à ces groupes.

**Conclusion**: Les priorités déterminées peuvent orienter la recherche axée sur le patient pour contrer la douleur chronique dans le but ultime d’améliorer les soins offerts aux personnes qui souffrent de douleur chronique.

## Introduction

Chronic pain is defined as a “distressing experience associated with actual or potential tissue damage with sensory, emotional, cognitive and social components” (p. 2420)^1^ that persists for more than 3 months, beyond the expected recovery time from an illness or injury, or that occurs in the context of ongoing tissue damage.^2^ Although it is often a disabling feature of chronic conditions such as arthritis,^3^ diabetes,^4^ cancer,^5^ cardiovascular disease,^6^ HIV,^7^ inflammatory bowel disease,^8^ and autoimmune diseases,^9^ chronic pain is now recognized as a disease in its own right.^10^

Chronic pain affects more than six million Canadians of all ages and is most prevalent among women, Indigenous peoples, and older adults.^11–13^ It negatively affects all dimensions of health-related quality of life and has a crippling financial impact.^14–16^ Between 35% and 50% of people living with chronic pain experience depression or anxiety,^17,18^ and chronic pain is associated with increased risk of suicide.^19^ Problematic use of substances is also a concern^19^; Canada has the second highest per capita rate of opioid prescribing in the world,^20^ and moderate quality evidence suggests that prescription of opioids for chronic non-cancer pain is associated with a 5.5% risk of opioid use disorder.^21^ Despite its enormous costs, until recently, less than 0.25% of all health research funding was directed toward chronic pain in Canada.^22^

In 2014, as part of its strategy for patient-oriented research (POR), the Canadian Institutes of Health Research (CIHR) called for proposals to establish collaborative national research networks focusing on chronic diseases. An integral part of POR is to include people with personal experience of a condition (generally referred to as “patients”) and their caregivers as partners throughout the entire research life cycle.^23^ This process includes involving patients in developing research priorities to ensure that public funds are allocated to projects that are viewed as useful, meaningful, and important by those who will be most impacted by their results.^24,25^

The James Lind Alliance (JLA) has developed a process referred to as a priority-setting partnership, which is designed to bring together clinicians and patients to identify treatment uncertainties or important questions about the treatment of health conditions that are not answered by current research.^26^ Using this process, research priorities have already been established for more than 50 illnesses,^27–30^ such as fibromyalgia^31^ and chronic kidney disease,^32^ but this has not yet been completed for chronic pain in general. The development of such a priority-setting agenda is imperative to guide national research efforts to ultimately improve chronic pain care and patient outcomes.

This article summarizes the process and results of a priority-setting project informed by the JLA to identify chronic pain research priorities in Canada, primarily from the perspective of people with lived experience of chronic pain and those who care for them, with input from clinicians, decision makers, and researchers.

## Material and methods

### Design and procedures

We used a modified priority-setting partnership approach informed by the JLA.^26^ The overall process involved gathering input from constituencies, including patients, clinicians, researchers, and decision makers from across Canada, to generate a comprehensive list of potential research questions. We then used a computerized Delphi survey,^33^ with four consecutive rounds to refine this list. The Delphi technique is a quantitative method that generates consensus from groups of people through a survey using an iterative process, with each round of survey responses being summarized and redistributed for a subsequent round.^34,35^ The Delphi process was deemed complete when the steering committee reached consensus that we had an appropriate number of research priorities that represented the range of suggestions brought forward by the participants throughout the process.

Our process diverged from the JLA approach in the following ways:

1. We did not limit questions to treatments but included questions focusing on the assessment and diagnosis of chronic pain, as well as questions related to the health care system more broadly as patient partnerships valued their importance. By using an open-ended approach, we ensured that any important questions falling outside treatment research were captured.
2. In accordance with the JLA, the panel that conducted the final priority-setting process was composed of only patients and clinicians. However, input from researchers and decision makers was sought during the generation of research questions. Researchers and decision makers also provided independent ranking of the importance of research questions for comparison; however, their ranking was not factored into the final selection of research priorities.
3. The JLA calls for a search to identify systematic reviews as well as ongoing trials to be conducted on all potential priorities to eliminate those questions where the answer is already known. This approach may demonstrate that a priority may not require immediate action.^26^ We chose a more parsimonious approach to complete the priority-setting process and leave the series of systematic reviews and meta-analyses as a separate and subsequent project.
4. Lastly, the JLA suggests that the final priority-setting process be conducted using face-to-face workshops with clinicians and patients. Instead, we used a computerized Delphi process^33–36^ to rank priorities until the final list was obtained. This allows for greater participation by a wider group of patients and clinicians.

The summary of the protocol for the project was reviewed by the chair of the Ottawa Health Science Network Research Ethics Board and was exempted from review following the UK Involve Statement, which articulates that involving people with lived experience in determining research priorities does not constitute research.^37^ Nevertheless, all potential participants were provided with information on the process and assurances of confidentiality because the Delphi did not ask for identifying features.

### Patient engagement mechanisms

**Figure 1.**
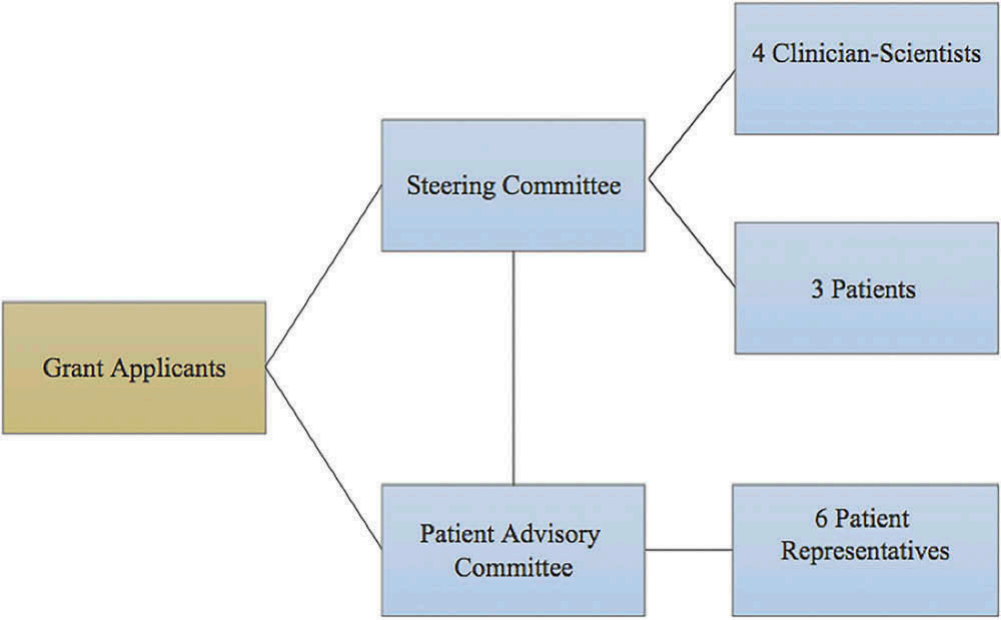
Project governance.

In accordance with CIHR’s strategy for patient-oriented research framework,^38^ patients were involved at all levels of research, including its governance. The steering committee included four clinician-scientists and three patients and was responsible for any major decisions about the study, taking into account the input from the patient advisory committee. The patient advisory committee, which was separate from the steering committee, included six patient representatives from various chronic pain associations (see Figure 1).

**Figure 2.**
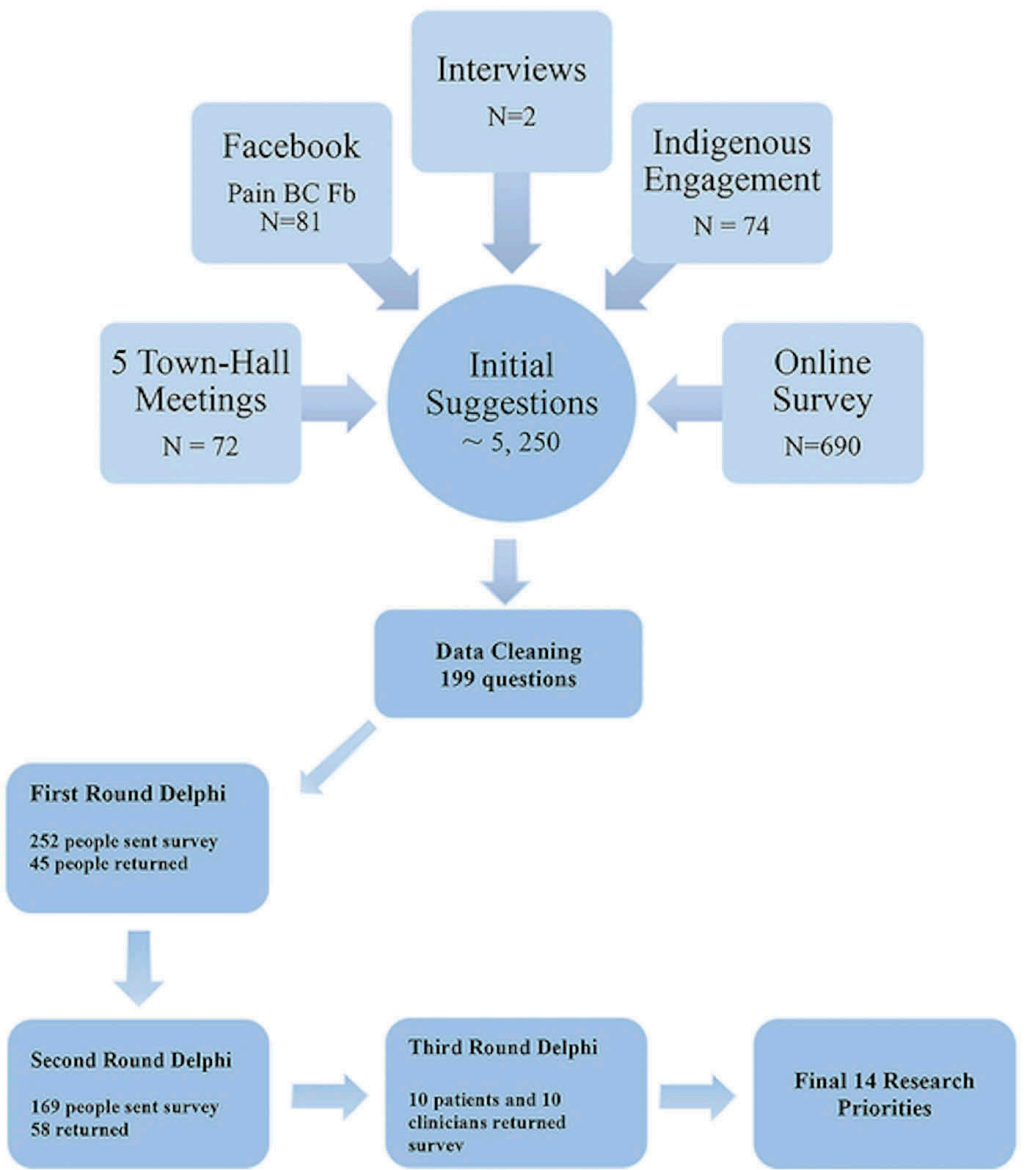
Project process.

Patients were offered different opportunities for engagement of varying intensities. Patients were co-investigators on the grant, provided input and co-wrote sections of the initial grant proposal, helped inform the research questions being asked, invited participation from people with lived experience from across the country, co-led focus groups, analyzed data, disseminated information, and prepared presentations of initial results to stakeholders. They are also co-authors on this article. One of the principal investigators identified as a person with lived experience of chronic pain and two people living with chronic pain worked as paid staff on the project. Key study materials were available in French and French-speaking patients were also involved as co-investigators and collaborators. A video detailing chronic pain and the need for a collaborative network was used for educational purposes and was subtitled in both languages to increase awareness about the project.^39^ This video featured patients, clinicians, and researchers to provide examples of their contributions.

We developed an independent process to ensure that Indigenous voices would be represented in this project. The rationale for this independent process considered that (1) chronic pain disproportionally affects Indigenous people; (2) some research among Indigenous people has been harmful; (3) health services for Indigenous peoples are organized differently across Canada; and (4) the engagement of Indigenous peoples as partners in research requires cultural sensitivity and experience.^40,41^ An Indigenous engagement lead and researcher (A.O.H.) with experience working with communities and participating in various committees led the process to address issues of importance.

### Participants

Participants were people with lived experience of chronic pain (e.g., patients, caregivers, and family members), clinicians, researchers, and decision makers with an interest in chronic pain. By reaching out to different groups and organizations, efforts were made to include people with different medical conditions (e.g., arthritis, Ehlers-Danlos Syndrome, multiple sclerosis), of different ages—including children, teens, and older adults—and who identified themselves at different points on the gender continuum (e.g., male, female, nonbinary). Except for the panel of clinicians and patients who conducted the final priority-setting round, we did not collect demographic characteristics. Nevertheless, details about participants in each of the phases of the priority-setting process are provided below. Due to the nature of the different engagement strategies used, an exact number of participants is not available but we estimate that a minimum of 1500 people participated (see further detail below in Figure 2).

#### Phase 1—Generating research questions

In the first phase of the project, we (1) conducted five in-person town hall meetings in Toronto (2), Ottawa (1), Charlottetown (1) and Halifax (1); (2) disseminated an online survey to people with lived experience, caregivers, and clinicians; and (3) used structured questions on Facebook to gather ideas about important research questions about chronic pain in Canada. The town hall meetings were co-led by different clinician-scientist–patient pairings who used principles of the nominal group technique^42^ to generate research questions to be included in subsequent phases of the priority-setting process. These town hall meetings included 83 patients. The online survey (see Appendix 1) included a series of 45 questions designed to elicit research questions pertaining to the prevention, assessment, diagnosis, treatment, and effectiveness of treatment of chronic pain as well as to chronic pain more broadly, including genetics, personalized medicine, and education. This online survey was active from September 20 to November 4, 2015. It was distributed widely through a snowball technique to all of the contacts of the steering committee and patient advisory group. This included two pan-Canadian chronic pain groups (Canadian Pain Coalition and Chronic Pain Association of Canada) as well as several provincial and local chronic pain or disease-specific groups; some were exclusively composed of patients, whereas others included clinicians and researchers. A total of 1481 survey responses were received, and of those, 690 contained suggestions for research priorities. The social media consultation on Facebook was led by Pain BC, a volunteer nonprofit organization dedicated to improving the lives of people living with chronic pain through education, empowerment, and innovation. Pain BC has over 6420 Facebook followers from across Canada, including people living in remote communities and Indigenous persons, and has experience in patient engagement using social media. Pain BC used its Facebook platform to ask a series of four structured questions to gather input from their followers (see Appendix 2). Two patients who learned about the project through the mechanisms described above also contacted the team to provide input directly via one-on-one conversations with two members of the coordinating team from Ottawa.

The Indigenous engagement lead conducted 74 one-on-one interviews (in person, via phone, or via e-mail) to gather input from clinicians and researchers from across Canada, many of whom identified as Indigenous, to generate a list of potential research priorities specific to Indigenous chronic pain research.

To ensure that everyone participating understood the project and what was being asked, we established a website^43^ with a description of the goals and objectives of the project and developed a brief 5-min video^39^ using infographics and featuring patients, clinicians, researchers, and the lead of the Chronic Pain Network team to describe the project. This video served as a knowledge dissemination tool and was used as an introduction to all of our activities. Direct viewing of the video on YouTube at the time of this article was 654 views. To increase dissemination of the project and increase participation, participants and patient group representatives leveraged Twitter to extend message and embedded links to the website^43^ and video on YouTube.^39^ We used these resources to disseminate information about the project to members of several patient organization (e.g., Canadian Pain Coalition, The ILC Chronic Pain, Ehlers Danlos Charitable Foundation, and Association Québécoise de la Douleur Chronique), disease-specific organizations (e.g., associated members of the ILC, Chiari Canada, Multiple Sclerosis, Ehlers-Danlos Syndrome Canada, Canadian Spondylitis Association), as well as members of the Chronic Pain Network.

#### Phase 2—First Delphi round

The first Delphi round consisted of a computerized survey circulated to patients and caregivers who indicated an interest in remaining involved in the project. The survey was framed as a way to guide the work of the Canadian Chronic Pain Research Network over the time frame of the chronic disease Strategy for Patient-Oriented Research (SPOR) grant and beyond. Specifically, we asked participants to indicate the importance of each question on a four-point numerical scale ranging from 1 to 4. The scale anchors were as follows: 4 (high priority for questions that the network should address as soon as possible); 3 (medium priority for questions that should be part of the Chronic Pain Research Network’s 5-year plan); 2 (longer-term priority for questions that should be considered for a longer-term plan for the Chronic Pain Research Network); 1 (not a priority; the network should disregard the question). The first round of the Delphi survey remained open from May 21, 2016, until June 13, 2016, and was distributed to 252 individuals who had expressed an interest in remaining involved in the project after Phase 1. Any questions on the survey list that received greater than 50% of the participants endorsing it as being a high priority were included in the second round of the Delphi survey (see Phase 4).

#### Phase 3—Integration of Indigenous chronic pain research priorities

We compared the list of priorities obtained in Phase 2 to the list of priorities generated through the Indigenous engagement process and consolidated these questions whenever possible (e.g., integration of the term “traditional healing practice” in the question focused on the effects of complementary and alternative medicine).

#### Phase 4—Second Delphi round

Patients, clinicians, researchers, and decision makers who had joined the Chronic Pain Network completed the second round of the Delphi survey and were given an opportunity to add questions. The survey remained open from August 16, 2016, to August 30, 2016, and was distributed to 161 SPOR participants who were instructed to rank questions with the same rating scale as the previous survey. Similar to the first round of the Delphi survey, any listed research questions that received greater than 50% endorsement from participants (clinicians and patients) as being a high priority were carried forward.

#### Phase 5—Final Delphi round

For the final phase of priority setting, a panel of ten clinicians from different health disciplines (medicine, physiotherapy, chiropractic, and naturopathic) and ten patients of different ages (mean age 48.3 years; range 27–71 years of age), genders, and chronic pain conditions was formed and this panel rated the list obtained in Phase 4. This final round of the Delphi survey remained open from September 23, 2016, to October 21, 2016. Only the questions that received greater than 50% agreement from the patients or clinicians were retained as research question priorities. These results were reviewed by the steering committee who determined that no further attempt to prioritize was necessary because the list of potential research questions was distinct. The final list of questions was reviewed by the steering committee and then organized thematically.

## Results

The initial consultations (Phase 1) yielded upward of 5250 entries, including comments, questions, and personal stories, and 252 people who participated in this phase indicated an interest in being part of subsequent phases of the priority-setting process. The list of questions was reduced to 199 items (by eliminating nonrelevant or duplicate items and consolidating similar questions). Forty-five people (out of a total of 252 potential participants; 17.9%) with lived experience of chronic pain (age rage 17–80 years of age, 62% women) responded to the first iteration of the Delphi survey to rate the importance of each of these 199 questions. This led to the selection of 38 questions with greater than 50% of the participants endorsing it as being a high priority included in the second round of the Delphi survey. Fifty-eight SPOR participants (out of 161 potential participants; 36.0%) responded to the next iteration of the Delphi survey: 21 (36%) clinicians, 41 (71%) researchers, 20 (34%) patients, and 5 (8.6%) decision makers (some people indicated that they belonged to more than one group, bringing the total percentage to greater than 100%). This process led to the identification of 23 questions that received greater than 50% endorsement as being a high priority by both clinicians and patients. Five questions that were considered high priorities by patients but did not reach this threshold for clinicians were retained to ensure that patients’ voices remained salient throughout the process. The final iteration of the Delphi survey led to the identification of 14 questions that were ranked as high priority by the panel of clinicians and patients.

The 14 questions identified were categorized under four broad themes: (1) improving knowledge and competencies in chronic pain; (2) preventing chronic pain and reducing associated symptoms; (3) patient-centered treatment of chronic pain; and (4) improving access to and coordination of patient-centered chronic pain care. The final list of questions is presented in Table 1.

**Table 1.** Top 14 questions for chronic pain research.

Improving knowledge and competencies in chronic pain
1. How can we increase knowledge and understanding of chronic pain both within the health care professions and within the community?
2. How can we improve competencies in chronic pain evidence-based practice among health care providers?
3. How do we address inaccurate beliefs about the use of opioids, cannabis, and other medications among patients, caregivers, health care professionals, and health policymakers to reduce underprescribing (barrier to access) and excessive or inappropriate prescribing (leading to higher risks of harm)?
Preventing chronic pain and reducing associated symptoms
1. What are the most effective interventions to prevent the development of chronic pain after acute injuries?
2. What are the biopsychosocial risk and protective factors in chronic pain, and in the presence of risk factors, what are some effective prevention strategies that could be implemented?
3. How can we prevent the development of and treat sleep, cognitive, social, or mental health problems among people living with chronic pain?
Improving patient-centered treatment of chronic pain
1. What tests/outcome measures should be used throughout pain treatments to measure progress and guide future treatment decisions and how can/should patient goals be taken into consideration in this context?
2. How can we best adapt treatments to take into account individual differences and does this improve treatment effectiveness?
3. Identify the key elements of a successful self-management program for chronic pain
4. What are the effects of various nonmedicinal options for the management of chronic pain, including but not limited to: exercise, functional movement therapy, traditional, complementary and alternative therapies?Which patients will benefit most from which treatment, what factors predict success, and do the effects depend on timing of intervention?
5. What are the effects of interdisciplinary treatments in contrast to other approaches such as pharmacological approaches and interventional approaches? Which patients will benefit most from which treatment, what factors predict success, and do the effects depend on timing of intervention?
Improving access to and coordination of chronic pain care
1. How can patients be better informed and more engaged in treatment decision making?
2. How can we increase capacity and reduce barriers to access to chronic pain diagnosis and treatment at different levels of the health care system?
3. How do we improve coordination of care and collaborations between health care providers working across settings of care, including providers of traditional and complementary and alternative medicine?

### Theme 1: Improving knowledge and competencies in chronic pain

There was broad consensus that increasing knowledge of chronic pain within both the health care professions and the community was an important priority. This was viewed as an essential step to reduce the stigma associated with living with chronic pain, as well as being an integral part of improving competencies in chronic pain evidence-based practice among health care providers, which was another identified research priority. A prominent topic was to find ways of addressing inaccurate beliefs about the use of opioids, cannabis, and other medications among patients, caregivers, health care professionals, and health policymakers to reduce underprescribing, which represents a barrier to access, as well as excessive or inappropriate prescribing, which is associated with harm.

### Theme 2: Preventing chronic pain and reducing associated symptoms

There was considerable interest in effective interventions targeting biopsychosocial risk factors for chronic pain and effective intervention strategies to prevent its development. Respondents also highlighted the importance of finding better ways of treating sleep-related, cognitive, or mental health problems in the setting of chronic pain to improve people’s quality of life and reduce disability.

### Theme 3: Patient-centered treatment of chronic pain

Most of the research priorities identified pertained to the assessment and treatment of chronic pain from a patient-centered perspective. This included interest in identifying which tests and outcome measures should be used to monitor treatment effectiveness and guide future treatment decisions while taking patient goals into consideration, identifying ways to adapt treatments to account for individual differences, and ascertaining whether this improves treatment effectiveness. Furthermore, there was consensus that efforts should be directed toward identifying key elements of successful self-management programs for chronic pain. Finally, there was consensus that research efforts should be directed toward investigating the effects of various nonmedicinal options for the management of chronic pain, including exercise, mindfulness, as well as traditional, complementary, and alternative medicine.

### Theme 4: Improving access to and coordination of patient-centered chronic pain care

The issue of access to and coordination of care was a predominant theme. Questions pertaining to increasing capacity and reducing barriers to access, chronic pain diagnosis, and treatment at different levels of the health care system and improving coordination of care and collaborations between health care providers working across settings of care were salient and received broad support for inclusion as research priorities. Under this theme was also the need for patients to be better informed and engaged in treatment decisions.

### Facilitating patient engagement in chronic pain research

Another significant result is that during our engagement cycles, 230 patients indicated an interest in remaining involved in chronic pain research and were offered entry into the Patient Engagement Committee of the Chronic Pain Network. Some have started to receive training on patient engagement and on becoming a patient partner.

## Discussion

This research priority setting process leveraged the work of well-established patient organizations and existing partnerships between researchers, clinicians, patients, and decision makers. This project engaged people living with pain and those who care for them to develop a chronic pain research agenda with the ultimate aim to improve care for all Canadians living with pain. Our vision was that the process and results could support the development and work of the Chronic Pain Network and that it would help guide chronic pain research efforts more broadly.

Fourteen research priorities across four themes emerged as important areas of focus for chronic pain research. The volume of questions aligns with most priority-setting projects, which often list between ten and 20 questions.^26^ We noticed similarities between priorities identified in our project with those of other priority-setting processes. For example, both our project and the CIHR Institute of Musculoskeletal Health and Arthritis 2016–Fibromyalgia^44^ priority-setting partnership identified personalized medicine, the role of nonpharmacological interventions, as well as education to encourage patients to take an active role in their own care.^44^ The fibromyalgia priority-setting process also identified the treatment and management of associated problems or symptoms (e.g., sleep and cognitive difficulties) to improve patients’ quality of life. We also found overlap with the anaesthesia and peri-operative care priorities; they identified finding ways to stop patients from developing chronic pain after surgery as their top priority^45^ and we identified the interventions to prevent the progression of acute pain toward chronic pain as a priority.

This project was not without challenges. The first challenge we encountered pertained to ethical regulations.^28^ Although the United Kingdom position paper^37^ clearly stipulates that engaging patients in identifying research priorities does not constitute research, our interactions with several ethics boards a well as provincial strategies for patient-oriented research units revealed a lack of consensus in this regard. With an initial 12-month timeline, this highlighted the importance for everyone involved to have realistic expectations regarding the realization of collaborative projects of this nature. It also highlighted gaps in the research ethics landscape regarding knowledge and policy about patient engagement.

The second challenge we encountered is that chronic pain is ubiquitous to many diseases and conditions. As such, many people offered suggestions that were specific to chronic illnesses such as fibromyalgia, Ehlers-Danlos Syndrome, or arthritis. We attempted to distill commonalities, as with the fibromyalgia priority-setting exercise, across these suggestions, irrespective of the conditions to which they were connected. However, it is possible that separate priority-setting processes would be warranted for each of these conditions.

The third challenge involved the engagement of vulnerable populations. We identified a number of groups that could benefit from focused engagement work to ensure that the research priorities established were congruent with their own respective priorities and that any other pertinent questions could be highlighted for the research teams to become aware of their importance. For example, though we anticipate that many of the priorities identified would be relevant to individuals living in long-term care and complex continuing care institutions, it is possible that the assessment and identification of chronic pain in this group might require different approaches (e.g., in the case of nonverbal individuals). In a similar manner, our initial Indigenous engagement process focused on clinicians and scientists whose work focuses on Indigenous health. Our process did not reach the community level, which would have required additional resources. This is not to say that Indigenous peoples were not included through our strategies, but future engagement work on chronic pain priority-setting for Indigenous peoples needs to take into account their diversity and traditional practices and ensure representation from communities across the country. Engagement of new Canadians, including refugees, was also lacking. Finally, although we did have representation of parents of children and teenagers living with chronic pain engaged in the process, this experience suggested that a separate priority-setting process might be more appropriate given their particular needs. For example, the role of parents in decision making about pain management or the transition and barriers between pediatric and adult chronic pain management settings might be worthy of significant attention to improve the care and quality of life of young people living with chronic pain. This did not emerge in the final priority-setting process, perhaps due to fewer pediatric-specific participants, and thus might emerge in a process targeted toward children, adolescents, and young adults/transitional youth.

Another important observation is that we had a low response rate from our social media consultation with Pain BC. This may be due to the fact that we only posted the questions once; therefore, the posts may not have been salient among other threads for members of the group who do not consult their social media regularly. It is also possible that members of the group who were interested in the project elected to complete the online survey instead of posting their ideas online. We also had low response rates to the first and second Delphi rounds; this is likely due to the fact that we sent these surveys more broadly to all people who had indicated an interest in remaining involved in the project (we had not asked people to identify specifically whether they wanted to complete additional surveys).

Finally, despite our aspirations to adhere to the values of inclusiveness, transparency, mutual respect, and adequate support to work together (co-build), we recognize some limitations. Lessons learned include paying more attention to the pre-engagement process by ensuring that sufficient time and resources be dedicated to clarifying roles, responsibilities, and expectations of all team members. For example, the team who developed and implemented this project was assembled very quickly; the steering committee and patient advisory were organized organically early on. Ideally, a formal process with calls for expression of interest would be used to establish working groups. Furthermore, given that patient engagement in research is relatively novel, pre-engagement should address these along with identifying and addressing any knowledge and skill gaps of partners. This would help ensure that participants work more effectively together and would help manage expectations (e.g., timeline for completion of consultation with or review by research ethics board). Nevertheless, even with the project’s limitations, more than 230 people indicated an interest in continuing to be engaged and have now been invited to be part of the patient engagement committee of the Chronic Pain Network, where the groundwork of building relationships, sharing knowledge, and working together on a common vision and goal is being done.

### Implications and future directions

The 14 identified priority areas represent broad directions for patient-oriented chronic pain research in Canada. Further work is required to ensure that these priorities are valid for different groups that are often underrepresented or marginalized in health research. For example, although we included teenagers and parents of young children in the process, it is possible that different high-level priorities would emerge as being key to improving the care of young people with chronic pain. The same observation may also apply to older adults. Further work is also needed to engage Indigenous communities as well as new Canadians who may have particular needs or challenges that were not identified as high priority by a sufficient number of people.

In parallel, systematic reviews are needed to evaluate the evidence available to inform each of the priorities identified. The results will facilitate identification of more precise questions to address knowledge gaps and spur the development of leading-edge research projects that can leverage the research infrastructure of the Chronic Pain Network and other relevant networks for efficiencies. This should continue to be done in partnership with knowledge users.

Finally, mechanisms must be developed to ensure that funding for chronic pain research in Canada is aligned with priorities identified through various priority-setting exercises that have been conducted. There is a commitment from the Chronic Pain Network to continue to use these priorities in the decision-making process to support new projects.

## Supplementary Material

1425980--Supplemental_Material.docx

## Appendix 1: Patient Engagement Survey

Chronic Pain Strategy for Patient-Oriented Research Network

We are a team of researchers, health care providers, and people living with chronic pain who are working together to identify research priorities related to chronic pain. We are asking patients with chronic pain, family members, caregivers, and health care providers what they think are the most important chronic pain–related questions researchers should be investigating. Your contribution in this project is completely voluntary. You may stop at any time, and we will not collect identifying information. The information we collect will be analyzed and published online so that researchers, clinicians, and people living with chronic pain know about the results of this project. If you choose, you will be given the opportunity to send us your contact information if you want to be kept informed about the Chronic Pain Strategy for Patient Oriented Research Network (Chronic Pain SPOR) at the end of the survey. If you have any questions about the project, you can contact Dr. Patricia Poulin or Dr. Jennifer Stinson at info@chronicpainmatters.ca. Thank you very much for your participation!

Please indicate if you are (choose all that apply):

**Table:**

□	Person living with chronic pain
□	Family member or caregiver of a person living with chronic pain
□	Health care professional
□	Researcher

Please list any chronic pain questions that you think should be answered by research. These questions can be focused on the prevention, diagnosis and treatment, or self-management of chronic pain. They can also be about how to live a good life with chronic pain, education about chronic pain, or anything else that you feel is important. If you find you are having difficulty thinking of specific questions, you can skip this page.

**Table:**

Question 1
Question 2
Question 3
Question 4
Question 5

Research in chronic pain covers many areas. In the next pages, we focus on different topics to help you to think about other possible research questions that are important to you.

**Prevention of chronic pain**

Research may help explain how and why chronic pain develops. It may also help us find ways to prevent chronic pain. There is a specific field of research that looks at risk factors for chronic pain—in other words, what things may make someone more likely to develop chronic pain. Studying these risk factors might help researchers make new discoveries that will help prevent someone from developing chronic pain in the future.

What factors do you think contribute to developing chronic pain?

(Please write as much as you can)

What questions do you think researchers should explore about the prevention of chronic pain?

(Please write as much as you can)

How important do you think this line of research is?

Please rate from 1 to 5 (1 = *not very important*, 5 = *extremely important*)

**Diagnosis and assessment of chronic pain**

Assessment of pain is an important part of diagnosing and treating chronic pain conditions because it helps us to more accurately understand your experience of pain and to figure out how effective treatments are in helping manage your pain. What way of measuring pain is most meaningful to you?

(Please choose one option)

**Table:**

∘	Using a scale of 1 to 10, with 1 being the least pain and 10 the most pain you could imagine?
∘	Using a scale with words like mild, moderate, and severe?
∘	Describing the pain’s impacts on your life and ability to do the things that matter to you.
∘	Other, please specify ______________________

If you tried a new treatment, what would you feel would be the best measure of its effectiveness?

(Please choose one option)

**Table:**

∘	Reduction in your experience of pain
∘	Increased ability to do the things that are important to you
∘	Improved sleep
∘	Improved mood
∘	Other, please specify ______________________

What questions do you think researchers should explore about the assessment and diagnosis of chronic pain?

(Please write as much as you can)

How important do you think this line of research is?

Please rate from 1 to 5 (1 = *not very important*, 5 = *extremely important*)

**Treatment of chronic pain**

People who live with chronic pain can sometimes be treated with medications, medical interventions (e.g., surgery), physical therapy, psychological therapy, as well as complementary and alternative medicine. The following section focuses on the treatment of chronic pain.

What questions do you think researchers should explore about the treatment of chronic pain?

(Please write as much as you can)

How important do you think this line of research is?

Please rate from 1 to 5 (1 = *not very important*, 5 = *extremely important*)

**Treatment of chronic pain—Medications**

What questions do you think researchers should explore about medications used to treat chronic pain?

(Please write as much as you can)

How important do you think this line of research is?

Please rate from 1 to 5 (1 = *not very important*, 5 = *extremely important*)

There are several medications commonly used today that were not originally designed for pain but that were found to have pain-relieving effects (e.g., some medications used to treat depression have also been found to help with chronic pain). Once the safety of these drugs was established, these treatments were studied to be used for pain. This is called drug repositioning.What questions do you think researchers should explore about drug repositioning?

(Please write as much as you can)

How important do you think this line of research is?

Please rate from 1 to 5 (1 = *not very important*, 5 = *extremely important*)

**Treatment of chronic pain—Medical interventions**

In some cases, interventions like surgeries or other procedures (e.g., injections) can be used to alleviate chronic pain. What questions do you think researchers should explore about medical intervention treatments for chronic pain?

(Please write as much as you can)

How important do you think this line of research is?

Please rate from 1 to 5 (1 = *not very important*, 5 = *extremely important*)

**Treatment of chronic pain—Non-drug strategies**

Medication is one of many treatments that have the potential to relieve pain. This next section focuses on different strategies that do not involve medications. Nonmedication treatment strategies can include physiotherapy, psychological approaches, complementary and alternative medicine, peer-to-peer support, ways to help you manage your own pain, as well as other techniques such as mindfulness meditation.

What questions do you think researchers should explore about non-drug chronic pain treatments?

(Please write as much as you can)

How important do you think this line of research is?

Please rate from 1 to 5 (1 = *not very important*, 5 = *extremely important*)

**Genetics and personalized medicine**

Researchers are looking to determine whether certain genetic markers have a role in chronic pain. These genetic markers might be used to predict who is more likely to develop chronic pain, who would benefit the most from certain treatments, who will have side effects from treatments or medications, and who is more likely to develop dependence on medications. Understanding this will help us to personalize treatments to each individual with chronic pain.What questions do you think researchers should explore about genetics and personalized medicine?

(Please write as much as you can)

How important do you think this line of research is?

Please rate from 1 to 5 (1 = *not very important*, 5 = *extremely important*)

Do you think people with chronic pain would be willing to get genetic testing as part of research studies?

**Table:**

∘	Yes
∘	No

Do you think people with chronic pain would be willing to get genetic testing to manage their condition better?

**Table:**

∘	Yes
∘	No

**Effectiveness of chronic pain treatments**

Researchers often look at a number of factors to determine whether a treatment is effective. In addition to reduced pain and improved sleep, mood, and function, researchers also consider:

What the side effects are and their impact on a person’s life.

What the risks of the treatment are.

How much the treatment costs.

Which factors do you think are most important when assessing the effects of treatments?

Please rank the options below in order of importance for you, with 1 being the most important.

(Please rank from 1 to 8)

**Table:**

	Reduction in your experience of pain	Increased ability to do the things that are important to you	Improved sleep	Improved mood	What the side effects are and their impact on a person’s life	What the risks of the treatment are	How much the treatment costs	Other
1	∘	∘	∘	∘	∘	∘	∘	∘
2	∘	∘	∘	∘	∘	∘	∘	∘
3	∘	∘	∘	∘	∘	∘	∘	∘
4	∘	∘	∘	∘	∘	∘	∘	∘
5	∘	∘	∘	∘	∘	∘	∘	∘
6	∘	∘	∘	∘	∘	∘	∘	∘
7	∘	∘	∘	∘	∘	∘	∘	∘
8	∘	∘	∘	∘	∘	∘	∘	∘

If you have ranked “other,” please specify below:

**Education**

There is a significant gap between what we learn from research and what we do in practice in health care settings.What do you think are the best ways to get what we learn from research into practice? Please rank the options below in order of effectiveness with 1 being your top choice.

(Please rank from 1 to 6)

**Table:**

	Educating health care providers	Creating specialty training programs for health care providers (e.g., a pain specialist, nurse, or physiotherapist)	Educating people in pain and their families	Creating alliances between researchers, health care providers and patients	Providing incentives (e.g., new fees for doctors to provide certain services) for better care	Other
1	∘	∘	∘	∘	∘	∘
2	∘	∘	∘	∘	∘	∘
3	∘	∘	∘	∘	∘	∘
4	∘	∘	∘	∘	∘	∘
5	∘	∘	∘	∘	∘	∘
6	∘	∘	∘	∘	∘	∘

If you have ranked “other,” please specify below:

**Summary**

Of all of the research topics included in this survey, what do you think are the most important for research to focus on? Please rank your top choices, with 1 being the most important.

(Please rank from 1 to 8)

**Table:**

	Prevention of chronic pain	Diagnosis and assessment of chronic pain	Treatment of chronic pain—Medications	Treatment of chronic pain—Medical interventions	Treatment of chronic pain—Non-drug strategies	Genetics and personalized medicine	Effectiveness of chronic pain treatments	Education
1	∘	∘	∘	∘	∘	∘	∘	∘
2	∘	∘	∘	∘	∘	∘	∘	∘
3	∘	∘	∘	∘	∘	∘	∘	∘
4	∘	∘	∘	∘	∘	∘	∘	∘
5	∘	∘	∘	∘	∘	∘	∘	∘
6	∘	∘	∘	∘	∘	∘	∘	∘
7	∘	∘	∘	∘	∘	∘	∘	∘
8	∘	∘	∘	∘	∘	∘	∘	∘

**General comments**

Please note that we will not respond to anything placed in this general comments box. If you would like to be in touch with us directly, please e-mail us at info@chronicpainmatters.ca.

(Please write down any other general comments)

**Next steps**

As we develop this chronic pain research network, we want to build more collaborations with people living with chronic pain and their caregivers. There can be many ways to be involved. One of them is to become part of a research team to help develop and implement research projects and to help share the results of these projects.Would you be interested in learning more about the type of chronic pain research that is being done by scientists and health care professionals across Canada? If so, what do you think would be the best way for us to do this? Choose all that you think would be helpful.

(Please select all that would apply)

**Table:**

□	Website
□	Facebook page
□	YouTube videos
□	Webinars
□	Twitter feeds
□	Other, please specify ______________________

Thank you so much for your time. We appreciate your involvement in helping us building a chronic pain research network for Canada!If you would like to be kept informed about this project, please fill in the attached form and send it along with the completed survey.

If you would like more information, please visit out website, http://www.chronicpainmatters.ca, or contact us directly at info@chronicpainmatters.ca.

## Appendix 2: Pain BC Facebook questions

Pain BC Facebook page questions:

1. Do you think we should explore genetic testing to manage pain?
2. What strategies have helped you cope with your pain?
3. What questions do you have about the assessment and diagnosis of chronic pain conditions?
4. What questions do you have about medications used to treat chronic pain?
